# *Drosophila* Functional Elements Are Embedded in Structurally Constrained Sequences

**DOI:** 10.1371/journal.pgen.1003512

**Published:** 2013-05-30

**Authors:** Ephraim Kenigsberg, Amos Tanay

**Affiliations:** Department of Computer Science and Applied Mathematics and Department of Biological Regulation, Weizmann Institute, Rehovot, Israel; Massachusetts Institute of Technology, United States of America

## Abstract

Modern functional genomics uncovered numerous functional elements in metazoan genomes. Nevertheless, only a small fraction of the typical non-exonic genome contains elements that code for function directly. On the other hand, a much larger fraction of the genome is associated with significant evolutionary constraints, suggesting that much of the non-exonic genome is weakly functional. Here we show that in flies, local (30–70 bp) conserved sequence elements that are associated with multiple regulatory functions serve as focal points to a pattern of punctuated regional increase in G/C nucleotide frequencies. We show that this pattern, which covers a region tenfold larger than the conserved elements themselves, is an evolutionary consequence of a shift in the balance between gain and loss of G/C nucleotides and that it is correlated with nucleosome occupancy across multiple classes of epigenetic state. Evidence for compensatory evolution and analysis of SNP allele frequencies show that the evolutionary regime underlying this balance shift is likely to be non-neutral. These data suggest that current gaps in our understanding of genome function and evolutionary dynamics are explicable by a model of sparse sequence elements directly encoding for function, embedded into structural sequences that help to define the local and global epigenomic context of such functional elements.

## Author Summary

A key challenge in functional genomics is to predict evolutionary dynamics from functional annotation of the genome and vice versa. Modern epigenomic studies helped assign function to numerous new sequence elements, but left most of the genome essentially uncharacterized. Evolutionary genomics, on the other hand, consistently suggests that a much larger fraction of the un-annotated genome evolves under selective pressure. We hypothesize that this *function-selection* gap can be attributed to sequences that facilitate the physical organization of functional elements, such as transcription factor binding sites, within chromosomes. We exemplify this by studying in detail the sequences embedding small conserved elements (CEs) in *Drosophila*. We show that, while CEs have typically high AT content, high GC content levels around them are maintained by a non-neutral evolutionary balance between gain and loss of GC nucleotides. This non-uniform pattern is highly correlated with nucleosome organization around CEs, potentially imposing an evolutionary constraint on as much as one quarter of the genome. We suggest this can at least partly explain the above *function-selection* gap. Weak evolutionary constraints on “structural” sequences (at scales ranging from one nucleosome to recently described multi-megabase topological domains) may affect genome evolution just like structural motifs shape protein evolution.

## Introduction

The molecular function of metazoan genomes has been studied extensively in the last decades, using progressively more extensive and sensitive techniques for profiling genome activity, modeling epigenomic organization and perturbing genome sequences. Genomes have been found to encode regulatory information affecting diverse functions, including gene expression, chromatin structure, recombination and replication. Despite this progress, only a small percentage of the e.g., fly, or human genome is annotated specifically with a well-defined molecular role. Comparative genomics and population genetics studies, however, estimate that 10–15% [1] of the human non-exonic genome evolves under natural selection. The gap between the two estimates is intriguing; what is the function of dozens of mega-bases with significant fitness contribution that lay in-between?

The genome of the yeast *Saccharomyces cerevisiae*, which is 200-fold smaller than the human genome, is packed with genes (covering 73% of the genome) leaving almost all of the intergenic genome to be in close proximity (89% within 1 kb or less) to a transcription start site or 3′ UTR element. Nevertheless, regulatory sequences with direct functional annotation (e.g. transcription factor binding sites) cover just a small minority of the implicated regulatory sequence. The remaining sequences have seemingly little encoding specificity and are typically viewed as wrappers for the “real” regulatory encoding or evolutionary leftovers of ancient functional sequences. Recent progress with the mapping and analysis of local chromatin structure next to transcription start sites showed that nucleosome packaging is correlated with transcription, and likely affecting gene expression and other biological processes. These data suggest that the functionality of regulatory sequences is modulated by the organization of nucleosomes around or over them [2], [3]. Interestingly, chromatin organization was found to be associated with the underlying sequence composition, and in particular, GC rich sequences were shown to package nucleosomes more efficiently than AT rich sequences (*in-vitro* and *in-vivo*) [4]–[9]. It was suggested that the local GC content (scale of 20 bp) in yeast evolves under selective compensatory dynamics [10]–[12] that stabilize hotspots of high AT content which are also low in nucleosome occupancy. In yeast, a major portion of the non-exonic sequences that are not directly coding for regulatory interactions is involved with (and selected for) defining the structure (i.e. nucleosome organization) of the genome.

Larger genomes encode regulatory information in a more dispersed and sparse fashion. The *Drosophila* genome is 10-fold larger than the yeast and 20-fold smaller than the human genome. The fly genome was one of the first to be screened genetically and sequenced completely [13]. Recently the genome was thoroughly profiled epigenetically [14], [15] and physically [16]. In spite of these efforts, the function of only a small portion of the genomic sequence has been described in detail, while a much larger fraction of more than 40–60% of the fly intergenic genome is estimated to evolve under selection [17]–[20]. While recent studies have suggested that fly nucleosome organization is also correlated with the local GC content [21], it is not clear to what extent fly genome sequences control and define the physical structure of the genome at the nucleosome level and beyond, and whether structural considerations can bridge the gap between evolutionary and epigenomic estimations of the genome's functional content [22].

In this study, we show that a substantial fraction of the fly genome is likely to evolve under “structural” constraints. We identify putative regulatory sequences by screening highly conserved elements using our recently published parameter rich probabilistic evolutionary model [23] and find that while conserved sequences are AT rich, they are surrounded by GC rich sequences showing high levels of nucleosome occupancy. Using a combination of analysis of divergence among fly species and population genetics in *Drosophila melanogaster*, we support the idea that about 25% of the fly non-exonic genome consists of sequences that embed highly conserved elements, and likely to evolve under weak selection that maintains localized GC content (i.e. in a scale of 20 bp). According to our model, a punctuated GC content increase around CEs is created and stabilized by the evolutionary dynamics of compensatory GC loss and GC gain substitutions. Considering structural and epigenomic constraints in models of genome evolution may contribute significantly to our understanding of genome function and how this function evolves.

## Results

### Non-uniform nucleotide composition and accelerated evolutionary substitution rate around fly promoters and DHS elements

**Figure 1:**
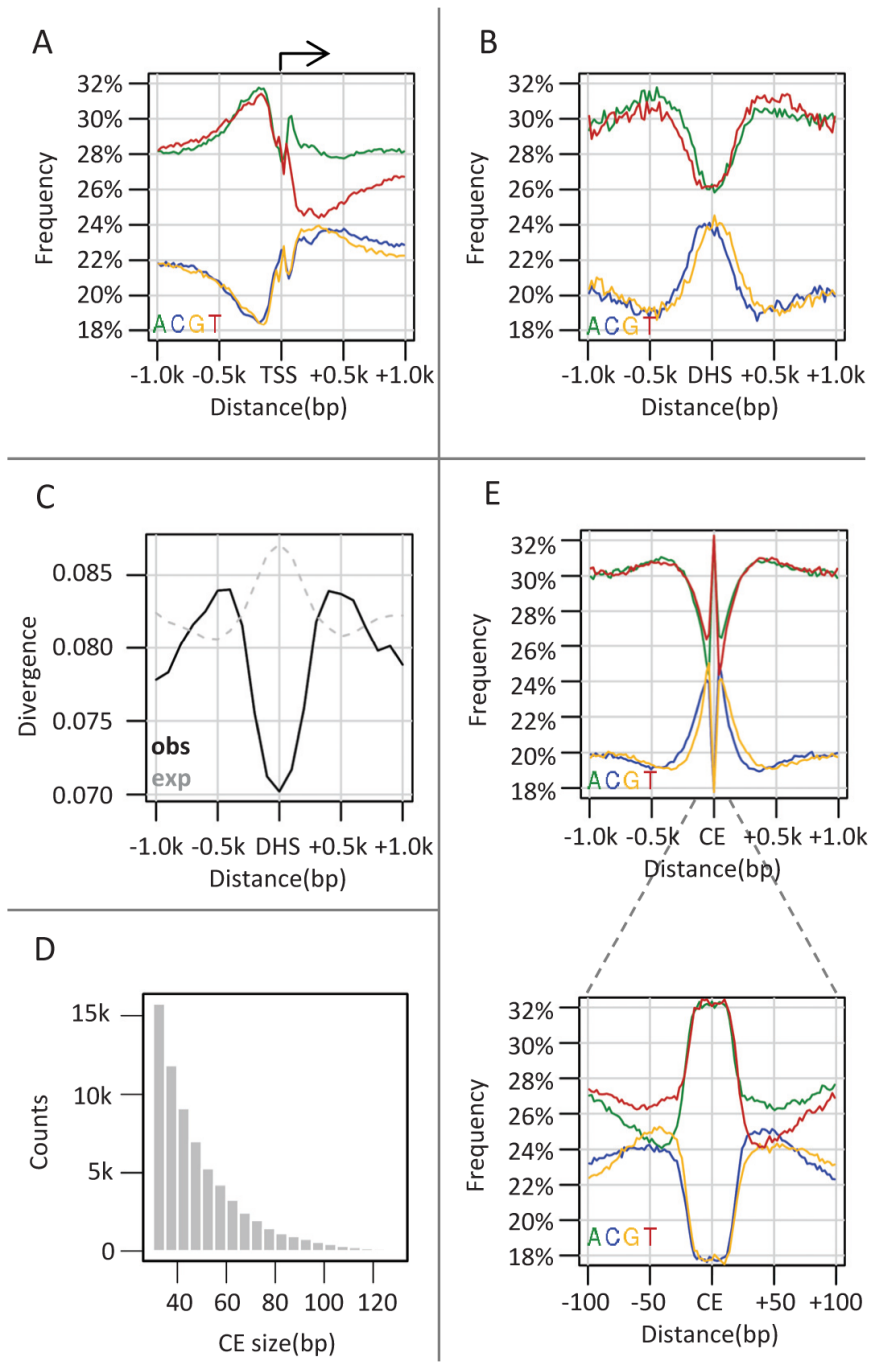
Punctuated GC elevation around conserved elements in flies. A) Nucleotide composition around TSS. Nucleotide frequency (y-axis) is depicted versus the distance to the nearest transcription start sites (TSS) (x-axis), obtained from UCSC genome browser. As previously reported, the frequency of A and T reaches a peak 200 bp upstream (left) to the TSS and decreases to the genomic average near the TSS. Also pronounced are AT asymmetry and GC asymmetry downstream to the TSS. B) Nucleotide composition around DHS. Similarly to A) but depicting nucleotide compositions relative to the center of the nearest DNase I hypersensitive sites (DHSs). DHS sites were defined as TSS distal (distance>1 kb) loci which are densely covered by DNase cutting sites (top 0.992 percentile, n = 2652) in at least one of four embryonic stages (stage number 5, 10, 11 and 14). C) Divergence around DHS sites. Shown are inferred (solid black line) and expected (given our substitution model, gray dashed line) substitution rates as a function of the distance to the nearest DHS sites. Rates were estimated from multiple alignments of 12 drosophila genomes (methods) using an evolutionary model that takes into account nucleotide composition biases and other context effects. The minor increase in expected substitution rate on DHSs (a consequence of higher GC content over DHSs), stands in marked contrast to the observed conservation pattern. D) Size distribution of conserved elements. CEs were identified at single base pair resolution as regions showing at least two-fold reduction in divergence compared to the expected rate. The size distribution of the inferred element is depicted, showing that most elements are smaller than 50 bp. E) Nucleotide compositions around conserved elements (CEs) are plotted over two distance scales (1 kb in top figure, 100 bp in inset). Regional (−300 to 300 bp) increase in GC content is observed in the larger scale, but the pattern is punctuated with high AT content (−20 to 20 bp) in the smaller scale.

Genome composition is associated with the underlying genomic functional annotation across multiple taxa, from yeast to mammals [24]–[26]. In particular, the distribution of GC content around gene promoters is known to be significantly different from the genomic background [25]. In flies, promoters show a well-known asymmetric skew in GC content (Figure 1A), with low levels upstream of the TSS (minima at −190 bp) and an increase toward the gene body. In contrast, DNase I hypersensitive elements (DHSs), which are strongly correlated with functional regulatory elements [27]–[30] show a regional elevation in local GC content (scale of 20 bp) peaking in the 200–400 bp around the DHS element center [31], [32] (Figure 1B, See Figure S1A–S1C for other classes of regulatory elements). To explore the evolutionary mechanisms underlying these well studied patterns we used a parameter-rich, context-aware evolutionary model [23], and estimated the substitution rates around DHS elements and promoters. As shown in Figure 1C, evolution within DHS elements is predictably slower than around them [33]. Perhaps more surprisingly, divergence rates around the DHS sites increase to peak levels around 500 bp from the center, before declining to background levels at ∼1000 bp. A similar evolutionary acceleration is observed ∼150 bp upstream to TSS (Figure S1D). These simple observations raised some intriguing questions regarding the evolutionary origins and functional significance of the sequences surrounding functional elements in flies, prompting us to explore the evolutionary dynamics within functional elements and around them in more detail.

### Conserved elements in flies are characterized by punctuated elevation in local GC content

The evolutionary model we used in order to characterize substitution rates in *Drosophila* genomes was designed to take full account of substitution rates variation among lineages and genomic contexts [23]. This algorithm learns evolutionary substitutions that are parameterized by flanking nucleotides and performs accurate inference of ancestral divergence within a phylogenetic tree encompassing 12 *Drosophila* species. This modeling and inference strategy ensures proper control for global biases in substitution rates at high or low GC content regions. We defined foci of genomic conservation as contiguous regions showing at least two-fold decrease in normalized divergence, identifying 67780 conserved elements (CEs) with an average size of 50 bp (and a standard deviation of 19.5, Figure 1D), covering in total ∼3% of the *D. melanogaster* genome (full list is available at http://compgenomics.weizmann.ac.il/tanay/?page_id=459). These conserved elements are enriched within enhancer associated transcription factors binding sites (Figure S1E), DHS and other epigenetically primed elements [31] (Table S1). Additionally, the evolutionary model allowed us to define CEs at a single base pair resolution and in a tissue independent fashion, something not possible using current epigenomic data. Remarkably, the high spatial precision led to a refined model of nucleotide composition around functional elements. Regionally elevated GC frequencies were observed as before, but this trend was punctuated by elements spanning 20–30 bp with a very high AT content (Figure 1E, Figure S1F). Furthermore, the symmetry of the compositions around CEs was skewed, with more A's and C's than G's and T's downstream the elements (see inset of Figure 1E). This observation significantly extends previous reports on associations between conserved sequences and local GC content [34]–[36], taking full advantage of the multi-species data and precision of the evolutionary model. In summary, by studying functional elements at a high spatial resolution, and using divergence statistics to define element boundaries rather than low-resolution epigenetic data, we show that the regional increase of local GC content around these elements is spatially anchored around foci of high AT content. This suggested that high GC content is a property of the genomic context of functional elements rather than a feature of the elements themselves, raising questions as to the possible evolutionary and functional mechanisms forming and using it.

### Epigenetic clustering shows that punctuated nucleotide profiles are common among multiple functional classes

**Figure 2:**
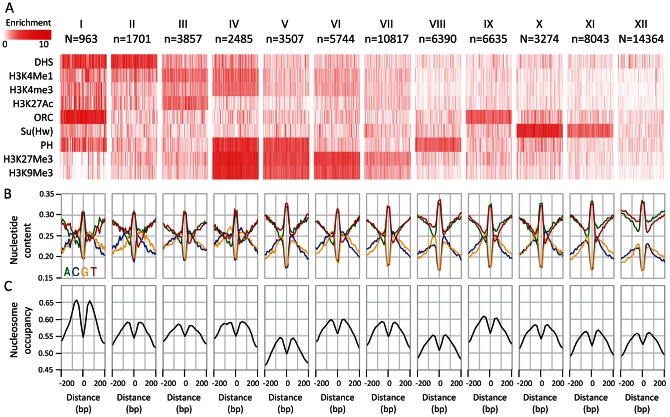
Epigenetic clustering of conserved elements. A) Classifying CEs according to their epigenetic context. Average enrichments (color coded, white-low, red-high) of selected epigenetic marks (rows) within CEs (columns) are depicted for groups of CEs clustered according to their epigenomic profiles. Due to size limitations, only 100 randomly chosen CEs are shown for each group. B) Average nucleotide compositions around CE clusters. While the basal GC content is variable between the epigenomic clusters, the punctuated GC elevation near conserved elements is pronounced in all of them. C) Nucleosome enrichment around CEs. Average nucleosome occupancy percentile (y-axis) around the CE centers is depicted for each cluster. A consistent depletion in occupancy over the CE center is observed for MNase-seq data of Weber et al. [69] in S2 cells.

The conserved elements we characterized above represent a heterogeneous set of functional and epigenetic behaviors. We wished to test if the remarkable nucleotide preferences around CEs are specific to particular epigenetic classes or observed globally. To this end, we clustered CEs given the association of the underlying loci with a representative set of epigenetic profiles (DHS, H3K4Me1, H3K4Me3, H3K27Ac, ORC, Su(Hw), PH, H3K27Me3, and H3K9Me3 (see methods)). We derived classes of CEs that were associated with several previously characterized epigenetic behaviors, including classes associated with promoter marks (Figure 2A, cluster i), enhancer marks (cluster ii,iii) Polycomb repressive domains (cluster iv–viii), origin of replication (cluster ix), and Su(Hw) insulator sites (cluster x,xi). We also identified a class of CEs that lacked enrichment in any of the markers (cluster xii). Detailed analysis summarized in Table S2 shows that this class also lacked enrichment in a comprehensive set of epigenomic markers [15], [27], [37], [38]. Importantly, regardless of the epigenetic context, and despite significant differences in the basal GC content (high at promoters and enhancers, low at uncharacterized elements), all classes of conserved elements exhibited the pattern of punctuated GC elevation that was demonstrated globally (Figure 2B). To verify that the nucleotide patterns we detected are observed at individual loci rather than being a result of some averaging effect, we clustered sequences at a single base pair resolution directly by their spatial GC profile in and around the CE (Figure S2). The clusters identified variations over the punctuated GC-elevation patterns (e.g. breaking symmetry to a particular side), but showed that only about 6% of the CE are lacking the punctuated GC elevation pattern. Importantly, analysis of nucleosome occupancy around CEs in the different classes unanimously demonstrated nucleosome depletion at the CE, with elevated nucleosome occupancy in the immediately surrounding regions (Figure 2C, Figure S2). This observation is consistent with previous reports on nucleosome occupancy distributions at specific epigenetic contexts [4], [21], [22], and suggests that coupling between sequence composition and nucleosome occupancy is a general phenomenon around CEs. The origins and functional implications of this coupling are not fully understood, but its extent raises hypothesis that the punctuated GC elevation pattern affects the accessibility and chromosomal structure around functional elements.

### Punctuated GC content at CEs is correlated with non-neutral GC dynamics

**Figure 3:**
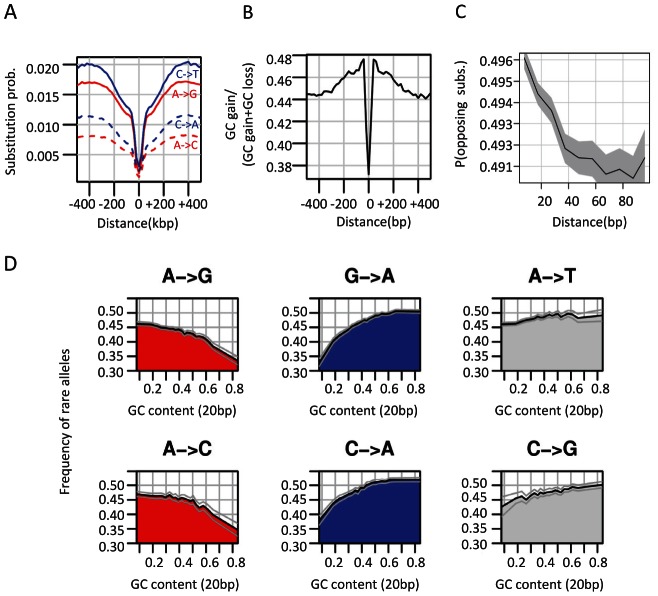
Non-neutral evolutionary dynamics around CEs. A) GC gain and GC loss rates around conserved elements. Shown are the average rates of different types of substitutions around CEs (see also Figure S3A). B) GC gain and loss balance. Plotted is the ratio of GC gain rate (combining all substitution types A/T→G/C) and total GC gain and loss rate at varying distance from CEs. C) Coupling between GC gain and loss. Using pairwise alignment of *D. melanogaster* and *D. yakuba*, we identified for each genomic distance (X axis) all pairs of loci within this distance that are both diverged between the two species. We then computed the fraction of such pairs that compensate an excess of GC in *D. yakuba* in one locus with an excess of GC in *D. melanogaster* in the other locus, out of all pairs of diverged loci. This non-parametric test shows that pairs of opposite GC changing substitutions are spatially more coupled as the distance between them decreases. Gray polygon represents binomial 95% confidence interval. For a parametric version of this test see Figure S3B. D) Frequency of rare alleles is correlated with GC content on a scale of 20 bp. We grouped SNPs according to mutation type and plotted the average frequency of rare allele p(0.01<minor allele frequency<0.05) for different levels of local GC content (20 bp). Binomial 95% confidence intervals are depicted as gray curves. See Figure S3D for analysis that is stratified for larger scale (200 bp) GC content.

Analysis of substitution rates around CEs showed that as expected, the highly conserved CEs are flanked by sequences with increasingly more diverged substitution rates (Figure S3A). We observed an increase in divergence toward a peak higher than the background level at around 400 bp from the CE, in a way analogous to the substitution dynamics around DHS elements and TSSs (Figure 1C, Figure S1D). Regional decrease in substitution rates toward the CE is observed for both GC gaining and GC losing substitution types (Figure 3A), but the ratio between the rates of these two processes recapitulated the punctuated nucleotide composition described above with an AT gaining regime at the CE, and a GC gaining regime peaking at 40 bp from the CE center (Figure 3B). This indicates that GC patterns around CEs are a result of a dynamic evolutionary balance rather than static conservation. To test if these non-uniform substitution dynamics represent a neutral mutational process or a more complex scenario, we analyzed spatial coupling between GC gaining and losing in the *Drosophila* genomes. We reconfirmed that on a scale of 1 kb, substitutions of the same type tend to cluster together (Figure S3B) [39]–[42], generating hotspots that increase their GC content during evolution on a given lineage. On a much finer scale (40 bp or less), the data suggest that this regional background regime is dominated by a stronger coupling between opposing substitution types (Figure 3C and inset of Figure S3B), such that G/C losing substitution are frequently matched by a nearby G/C gaining substitutions (and vice-versa). Coupling of opposing substitutions is predicted by theoretical models of compensatory evolution in which weak selection pushes forward substitutions that compensate for prior fitness reduction caused by proximal substitutions [12], [43], [44]. We conclude that regions flanking CEs evolve under non neutral evolutionary dynamics that are potentially stabilizing variable local GC content (on a scale of ∼20 bp) through compensatory coupling between GC gaining and losing substitutions. The non-neutrality of this regime (reminiscent of the compensatory dynamics in yeast promoters [12]), raises the possibility that sequences embedding fly CEs are functionally constrained to preserve some structural genomic role.

### Local GC content is associated with allele frequency of G/C gaining and G/C losing SNPs

Within-species quantification of frequency of rare alleles can provide a robust indication for a non-neutral evolutionary regime [45], less affected by variability in mutation rate than quantification of divergence rate between species. To further examine the evolutionary dynamics at loci with high or low local GC content (and in particular in sequences embedding CEs), we analyzed allele frequencies at 1.3 million *D. melanogaster* SNPs [20], [46]. We classified SNPs according to their mutation type (GC gaining/losing, transition/transversion), and stratified the analysis given the local (20 bp) GC content around the SNP locus. As can be seen in Figure 3D, the frequency of rare alleles in *D. melanogaster* is strongly correlated with the local GC content in a mutation-type dependent fashion. High frequency of rare alleles is observed when losing a G/C in a GC rich element, or when losing an A/T in an A/T rich element. Since previous studies had suggested that GC polymorphisms are associated with GC content on larger scales than those analyzed here [47] we stratified the allele frequency analysis using GC content estimation at the 200 bp scale (Figure S3C). We observed that localized GC content correlates with allele frequencies, even when conditioning for regional variation in GC content, pointing toward effects that work on a scale of few dozen bp, such as those characterized around CEs (Figure 1E). Taken together, divergence and polymorphism data are both consistent with a model of selection on local GC content in flies, which is particularly pronounced in the immediate context of CEs, and is correlated with chromosomal structure but not with any other known coding or TF specificities.

### Microsatellites are uniformly enriched near conserved elements

**Figure 4:**
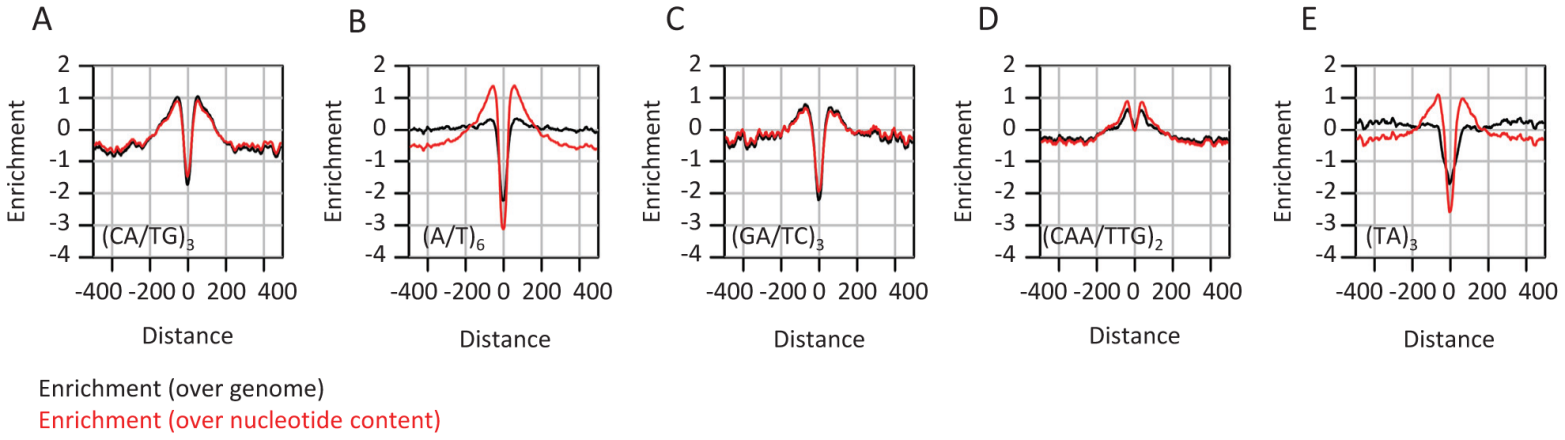
Enrichment of microsatellites in the proximity of conserved elements. A–E) Shown in black are distributions of enrichment (log2 of the ratio between the frequency at a certain distance from a CE and the global average) computed for various tandem repeats and motifs around CEs (black). Shown in red are similar statistics, but this time normalized according to the nucleotide compositions of the corresponding distance around CEs (i.e. dividing the frequency of each k-mer by the product of its nucleotides' frequencies). The data suggest remarkably uniform enrichment of tandem repeats around CEs.

The analysis discussed above focused on evolutionary dynamics at the nucleotide composition level. This approach provides the simplest description of the structure and evolution of sequences embedding CEs in flies, but it may be biased or incomplete if more complex sequence elements are evolving non-neutrally around CEs. Indeed, several frequent sequence motifs, most notably poly-A/T tracts and CA repeats were suggested before to modulate genome structure through regulation of epigenomic organization [48]–[52]. In some cases, (e.g., yeast poly-A element), this idea is supported with perturbation experiments [2], but in other cases the role of low-complexity sequence elements is difficult to support directly. We performed exhaustive screening of the k-mer spectrum (Table S3) around CEs, aiming at the identification of sequence patterns that are distributed non-uniformly around the elements center. Although evolutionary unstable (Figure S4A–S4D), some microsatellite elements like CA repeats are abundant near conserved elements (Figure 4, black curves). Other short repeats (e.g (TA)_n_, poly A/T tracts) were not found to be frequent in these loci. Despite the seemingly specific enrichment of CA repeats in the context of CEs, normalization of microsatellite frequency given the observed nucleotide compositions around CEs (Figure 4, red curves) indicates that short repeats ((CA)_n_, (TA)_n_, (CAA)_n_, (A/T)_n_) are in fact enriched around CEs at remarkably similar levels. This suggests that enrichment of simple repeats is a general property of the sequences flanking CEs, and is unrelated to the properties of particular dinucleotides. Several mechanisms may underlie this phenomenon, including higher rates of short insertion and deletion mutations in regions with an active chromatin structure [53], or alternatively, reduction of the efficiency of purifying selection next to functional element sequences (genetic draft) which is leading to higher sensitivity to microsatellite infiltration [54].

### Local GC content correlates with allele frequencies in mammalian genomes

Finally, we surveyed the association between local GC content and allele frequencies in human and mouse, aiming to test if similar trends as those defining the embedding of CEs in flies may affect mammalian genome structure. In mammalian genomes functional elements are sparser, and the estimated overall evolutionary constraint is considerable [1], [55] but not as global as it is in flies. Moreover, selection-like effects, which are frequently associated with biased gene conversion [41] are affecting the evolutionary dynamics of large scale (>1 kb) GC content distributions. As shown in Figure S5, the distributions of rare allele frequencies in human (4.05 million SNPs, Figure S5A) and mouse (63.8 million SNPs, Figure S5B), are highly dependent on both regional and local G/C content. Similarly to fly, GC losing minor alleles are rarer at elements with high local (20 bp) GC content, independently of the regional (200 bp) GC content around them. The opposite trend is observed for GC gaining SNPs. One can hypothesize that structural constraints such as those suggested by the *Drosophila* data are also active around regulatory elements in mammalian genomes, although their intensity may be decreased due to the sparser genomic structure. Indeed, recent reports on the evolutionary dynamics near mammalian nucleosomes [56] support this idea.

## Discussion

### Base pair resolution analysis of evolutionary conservation and nucleotide composition in *D. melanogaster*

We have combined inference of context-dependent substitution rates in *Drosophila* species, with epigenomic data and sequence analysis to show how highly localized, 30–70 bp conserved elements (CEs) in the non-exonic fly genomes are embedded into the genomic sequence in an organized and structured fashion. We showed that CEs are typically located within a few hundred bp of elevated GC content regions, but that the CE itself is punctuating this pattern with a sharp increase in AT content. The substitution patterns supporting this highly non-uniform organization are modulating the processes of GC gain and GC loss such that the immediate flanking of the conserved elements has GC gaining substitutions in relative excess. We discovered local compensatory coupling of GC gaining and GC losing substitution on a scale of up to 20–40 bp (and in contrast to the previously reported regional clustering of substitution types that we observed on a scale of about 100–1000 bp). Furthermore, analysis of polymorphic sites identified a remarkable association between highly local (20 bp) GC content and the allele frequencies of GC gaining and GC losing mutations, a phenomenon that is recapitulated in analysis of human and mouse data.

GC content is amongst the most thoroughly explored and modeled genomic phenomena. It is known to be correlated with recombination rates [57], replication time [58], and a plethora of other proven and hypothesized effects. Importantly, our observations here, which target the highly localized organization of regulatory factors and nucleosome within a scale of few dozen base pairs, are significant even when considering the larger scale GC content trends studied before. In conclusion, short non-exonic conserved sequence elements in flies are focal points of non-random and non-uniform genomic organizational units that may cover as much as one quarter of the fly genome (Figure S5C). The evolutionary process that gives rise to these organizational features is unlikely to be neutral, implicating a large fraction of the genome with natural selection and weak, but significant functionality.

### Neutral and non-neutral dynamics near conserved elements: The case of microsatellites

While multiple lines of evidence (divergence patterns, allele frequencies, compensatory coupling) suggested that the evolution of sequences surrounding functional elements in flies is affected by selection, complete characterization of the evolutionary dynamics in these regions is still challenging. For example, powerful selection on the functional elements themselves may result in recurrent selective sweeps or other effects originating from the linkage between the elements' cores and their sequence contexts. These may alter the divergence and polymorphism patterns around the cores of functional elements. Furthermore, DNA replication and repair processes may be affected by the element's polarized chromatin structure, resulting in higher or lower rate of mutation, or in variation of the mutation spectrum. Our data suggest that these considerations play a major role in at least one aspect of the sequence composition around functional elements, namely, the documented enrichment of (CA)_n_ and other microsatellite repeats [48]–[52]. We showed that the specificity of microsatellite abundance around CEs (e.g., many (CA)_n_, but few (TA)_n_) evens out when considering the nucleotide composition in these regions. Instead of enrichment for specific repeats, that could have suggested these elements have specific functional roles, we observed consistent increase in the frequency of all microsatellites families. In contrast to the patterns of punctuated GC content increase discussed above, CE-linked repeat elements could not be associated with signatures of non-neutral evolution. We therefore hypothesize that the accumulation of microsatellites around CEs is explained by either an increased mutation rate, or decrease in the efficiency of purifying selection due to the powerful evolutionary dynamics and epigenomic characteristics of functional elements.

### Functional and structural organization of metazoan genomes

**Figure 5:**
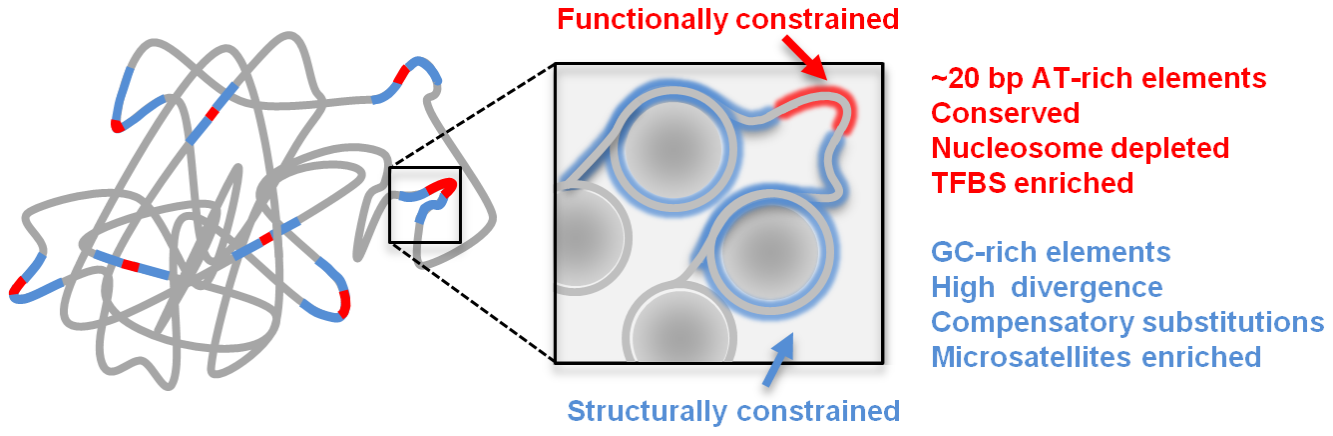
Schematic model of genome structure and function. Conserved elements (red) are surrounded by structural sequences (blue) which are packaged by nucleosomes (gray circles) and crucial for maintaining the physical structure associating the CEs into functioning chromosomes.

Genomic sequences are the outcome of a multi-faceted set of requirements, molded into form through an indirect and complex evolutionary process. Sequences contain *codes* that facilitate their functions, but in order to transform these codes into physical molecules, sequences must also contain structural elements that allow the codes to be interpreted [51], [59], [60]. For example, complementary constraints on function and structure are well known to affect the form of protein coding sequences [61], and these can consequentially be decomposed into structural motifs and functional hotspots. In the study of genome structure and function such considerations are still largely unexplored. Nevertheless, the accumulating evidence on local (e.g. nucleosome organization, [4], [21], [62]) and global (e.g. chromosomal conformation, [16], [63]) structural features of metazoan genomes are showing that while only a small fraction of the genome is directly coding for classical functions (protein coding, regulatory elements), the large remaining fraction of the genome is crucially important for proper function. As shown here, this view is now supported by the sequence composition and evolutionary dynamics detected at sequences embedding functional elements in *Drosophila*. We hypothesize that classical enhancer elements, which encompass several hundred base pairs and are capable of driving tissue specific transcription or repression given appropriate trans- factor activity, may contain one or few small (order of 10 bp) *code* words capable of recruiting such factors, embedded into larger and more diffused sequences that support the epigenetic organization of the enhancer region. Such epigenetic organization may involve local nucleosome occupancy and stability, as well as more complex interaction of the enhancer with remote elements (as in promoter-enhancer interaction [64], [65], or contacts between Polycomb response elements [66]). The resulting model for genome structure and function (Figure 5) can explain many enigmas of modern genomics, including the discrepancy between the estimated intensity of selection on large genomes and the fraction of properly annotated sequences, and the highly non-uniform sequence content distributions (e.g. GC content, simple repeats) in different genomic regions. We hypothesize that by viewing the genome as a physical molecule, not only the container of abstract information, we will be able to understand and interpret normal or aberrant gene function at new levels, especially when combining sequence analysis with extensive linear and three-dimensional epigenomic data.

## Methods

### Sequence and epigenomic data

Multiple alignments (dm3 version), gene annotation data and annotation of repetitive elements were downloaded from the UCSC genome browser [67]. DHS data of five embryonic stages [27] were downloaded from SRA (SRA012889). Replicates were merged and mapped to dm3 using bowtie [68]. modEncode [15] Chip-Seq tracks were downloaded from modEncode website. Nucleosome data [69] were downloaded from GEO (GSM539582). We also used polycomb group chip-chip data of Schuettengruber et al. [37]. Experimentally validated transcription factor binding sites were downloaded from the REDfly website [70]. Genomic loci which are annotated as exonic or repetitive (defined by UCSC genome browser as LTR, LINE, RNA, DNA, satellite or unknown) were excluded from further analysis to minimize biases associated with genome alignments and short read mapping.

### Normalization of epigenomic marks

The epigenomic data was smoothed by averaging the data values in each non-overlapping adjacent twenty base pairs. DHS data were then smoothed in 200 bp windows (with overlaps). All data were then normalized using the following non parametric transformation:
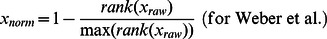


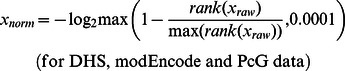



### Peak definitions

DHS sites were defined as TSS distal non exonic loci (TSS distance>1000) of DNase I levels higher than the 0.992 percentile in at least one of the embryonic stages (stage number 5, 10, 11 or 14). Note that this set was defined to represent the distribution of active regulatory regions, but is not intended to be generating an exhaustive set of elements. We used only TSS distal to DHS solely for the purpose of comparison between DHS and TSS patterns. dRing peaks were defined as non-exonic loci with dRing levels higher than the 0.995 percentile in S2 or BG3 cell lines. H3K27Ac peaks were defined as non-exonic loci with H3K27Ac levels above the 0.995 percentile in one or more embryonic stage (E0–4 h, E4–8 h, E8–12 h, E12–16 h, E16–20 h, E20–24 h).

### Screening of conserved elements

We used our recently described context-dependent substitution model to compute conservation scores at one bp resolution across the *Drosophila* clade (http://compgenomics.weizmann.ac.il/tanay/?page_id=169). As described in Chachick et al [23], our algorithm learns substitution probabilities 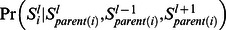 for each type of point substitution (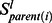
→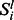), flanking nucleotides (
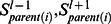
) and lineage (parent(i)→i). The algorithm then performs ancestral inference and computes accurate posterior probabilities for single nucleotide and triplets of nucleotide at each genomic position and for combinations of ancestral triplets in parent and child species. Based on these posterior probabilities, we computed the number of substitutions that occurred at each genomic locus (integrating over all of the *Drosophila* lineages) and the number of substitution expected given the probabilistic model. By comparing observed and expected number of substitutions in overlapping windows of 20 bp we estimated the degree of evolutionary conservation while controlling for biases associated with variable divergence rates in different nucleotide contexts.

Conserved hotspots were defined as non-exonic loci where the estimated number of observed substitutions is two-fold smaller from the expected number of substitutions. Following expansion of each hotspot by 10 bp to each direction we merged resulting overlapping intervals. Conserved elements (CEs) were then defined as unified genomic intervals spanning over 30 bp. Average profile of observed and expected divergence scores around regulatory elements are shown in Figure 1C and Figure S1D.

### Plotting sequence profiles around DHS, CEs, and other peaks

Given a loci set L we define, for each genomic location i its minimal distance from L as:
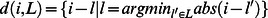



Nucleotide composition profiles of Figure 1A, 1B, 1E; Figure 2B; Figure S1A–S1C, S1F; and Figure S2 were generated by measuring the nucleotide frequencies of non-exonic sequences as a function of d(I,L), where L represented the centers of loci of each sets (DHSs, CEs, or other peaks). Note that this procedure retains the chromosomal polarization of these loci and assures that each nucleotide will be counted once.

### Clustering by epigenetic data

Enrichment scores of several epigenetic marks over CE were calculated by taking the average enrichment values of 9 epigenetic marks: DHS (embryonic stage 10), H3K4Me1 (E16–20, GSM401403), H3K4Me3 (E16–20 h, GSM400658), H3K27Ac (E16–20 h, GSM401401), ORC (s2, modencode_2573), SuHw (s2, SU(HW)-HB.S2 of modEncode), PH (4–12-h) [37], H3K27Me3 (E16–20 h, GSM439444), H3K9Me3 (E16–20 h, GSM439453). CEs were clustered using k-means (Euclidean distance) into 12 clusters. To ensure that the results of Figure 2 are not sensitive to the choice of epigenetic makers, clustering was performed by chip profiles of different embryonic stages, tissues, histone modifications and TFs. This yielded similar results, consisting of clusters representing the same epigenomic classes.

### Clustering by sequence data

To investigate the distribution of nucleotide compositions around CEs at high resolution, it was first important to neutralize the effect of the highly abundant (WS)_2_ microsatellites (e.g. (CA)_n_, (GA)_n_). These sequence elements generate strong signatures that were studied separately (see Figure 4). After extracting sequences within a distance of 100 bp from centers of all CEs, each two base-pairs were transformed to {0,1,2} by the number of G and C, thereby averaging out the signature of repeats with a 2 bp periodicity. This procedure yielded 67780 vectors of 100 numbers between 0 and 2, which we clustered into 20 groups using a standard k-means algorithm with a Euclidean metric. The clustering did not yield sets which were enriched with 3-bp repeats, and thus it was not needed to further smooth the sequences.

### Compute spatial trends of GC gain and GC loss

We used our context aware and lineage specific evolutionary probabilistic model to estimate posterior probabilities for individual nucleotides 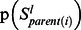 and pairs of parent-child nucleotides at each genomic locus 
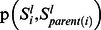
 [71]. For a given set of loci L, (e.g. all loci of a certain distance from CE), we define:
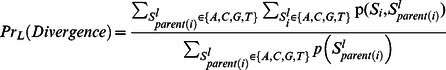


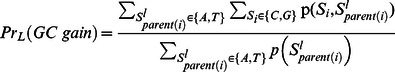


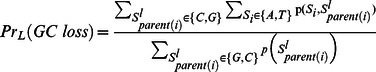



Depending on the analysis, loci were further grouped to compute the evolutionary trends around them.

### A non-parametric test for spatial association between substitutions

Aligned sequences of *D. melanogaster* and *D. yakuba* were extracted from the fly 12 species multiple alignment, omitting loci of insertion and deletion between these species. We computed the GC content difference at each locus (aligning two nucleotides) as follows:
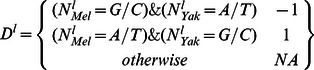



We then selected a distance parameter d, excluded loci of NA values, and computed the coupling score for d as:
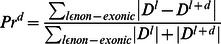
using only loci for which the range [l,l+d] had at most four bp gaps and further restricting loci to e.g., sequences flanking CEs. We estimated confidence intervals for observed coupling scores (Figure 3C) assuming a binomial distribution (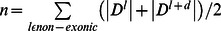, α = .05).

### A parameter rich test for compensation between substitutions

To validate the spatial association between GC gain and GC loss substitutions, we used posterior substitution probabilities, computed as outlined above, and focused on the dynamics in the *D. yakuba* lineage. Non exonic genomic sequences were screened for loci of GC gain and GC loss on the *D. yakuba* lineage by the following criteria:
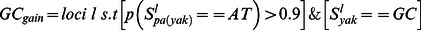


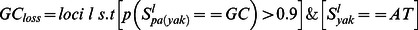



We then estimated the rate of different substitution types (gaining GC or losing GC), conditioned on the existence of GC gain or loss event within a distance (one sided) of at most d nucleotides (Figure S3B):
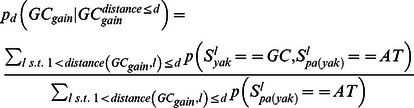


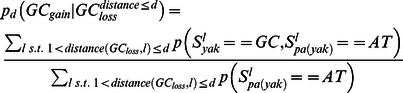


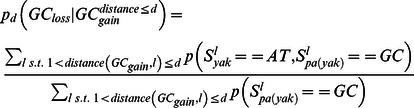


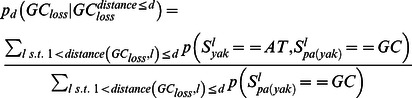



### Fly SNPs analysis

Fly SNP data were downloaded from DGRP website (http://www.hgsc.bcm.tmc.edu/projects/dgrp/freeze1_July_2010/). This dataset [20] consist of genomic sequences of 162 inbred *D. melanogaster* lines. SNPs of more than two distinct alleles were excluded. To minimize the contribution of sequencing errors, we also excluded very rare SNPs (p<0.01) and SNPs with low average coverage (<10). We defined the major and minor alleles of each locus by their relative abundance in the population. Non exonic and non-repetitive SNPs were divided into 20 bins by the local GC content (20 bp) of their genomic location according to the reference genome.

Frequency of rare allele of N→N′ SNP is defined as (N, N′ may be any non-identical nucleotides):
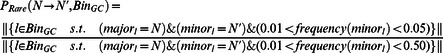



For further control, SNPs were divided into cubic bins by the local GC content of two scales: 20 bp (20 bins)×200 bp (5 bins)
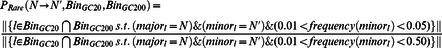



### Mammalian SNPs analysis

Human SNP data [72] were downloaded from http://hapmap.ncbi.nlm.nih.gov/downloads/genotypes. Mouse SNP [73] data were downloaded from http://www.sanger.ac.uk/resources/mouse/genomes/. SNPs that fell within repetitive elements (as defined by UCSC genome browser) were excluded. We defined the major and minor alleles of each locus by their relative abundance in the population. Non-exonic and non-repetitive SNPs were divided into cubic bins by the regional GC content of two scales: 20 bp (5 bins)×1 kb (5bins).

Frequency of rare allele of a N→N′ SNP is defined as:
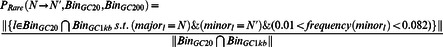



## Supporting Information

Figure S1 Fly regulatory elements are characterized by increased GC content and non-uniform evolutionary dynamics. A–C) Nucleotide composition around regulatory elements is shown versus the distance (x-axis) to the center of the nearest peak of A) H3K4Me1, B) H3K27Ac and C) dRing. The data showed that GC content is enriched over a region spanning ∼0.5 kb around regulatory elements of different classes. D) Increased substitution rates in fly promoters. Shown is substitution probability inferred from analysis of 12 *drosophila* species as a function of distance from the nearest TSS. While the divergence is lower near TSSs (more conserved), the 1 kb region upstream to TSS is highly diverged comparing to the background rate of evolution, reaching the maximum rate ∼150 bp upstream to TSS. E) Enrichment of transcription factor binding sites in CEs. Enrichment of REDfly experimentally validated transcription factor binding sites [70] is shown versus the distance from CE centers. Enrichment score is defined as the log2 ratio of the number of observed REDfly sites within a given distance range and the expected number of sites under uniform genomic distribution. F) Punctuated GC pattern in DHSs with weaker conservation. Shown are nucleotide frequencies versus the distance (x-axis) from the loci of minimum divergence within 1587/2652 DHSs that lack a CE given our current thresholds. To compute the pattern, we centered all elements on the positions with highest conservation score. Although these loci were not classified as CEs according to a stringent threshold, their punctuated GC pattern is significant.
(TIF)

Figure S2 Classes of CE sequence compositions support the universality of the punctuated GC elevation pattern. CEs were clustered using k-means given their nucleotide composition patterns (Methods). Shown are nucleotide compositions and nucleosome occupancy profiles (y-axis) versus distance to the center of the nearest CE (x-axis) for 20 clusters inferred. We note that the clusters' nucleotide composition profile is varying in basal GC content and in symmetry, but that with the exception of one cluster (XI) all groups of CEs showed GC elevation in their proximity.
(TIF)

Figure S3 Evolutionary dynamics are associated with local GC content. A) Inferred substitution rates around CEs. Shown are substitution rates of all mutation types versus the distance from the nearest center of conserved element averaged over the *melanogaster* group lineages. Solid lines are color coded according to the substitution types A→C (black), A→G (red), A→T (green), C→A (blue), C→G (cyan), C→T (magenta). Dotted lines represent the complementing mutations. B) Association between GC gain and loss. We quantified the spatial coupling between GC gain and loss substitutions over the *D. yakuba* lineage, by estimating the rate of one type of substitution (GC gain in black, GC loss in red), conditioned on the existence of another type of substitution at a certain distance from it (X axis, Methods). The ratio between the two types of conditionings provides indication to the extent of coupling showing opposite trends for GC losing and gaining substitutions. Strong compensatory coupling was observed locally (at distances of less than 20 bp, see inset), while on the long range (e.g. >2 kb) we observed coupling of GC losing events (i.e. red curve is below 1). C) Stratification on regional GC content shows that fly allele frequency is strongly associated with local GC content. Shown is frequency of rare alleles (y-axis, see methods) against the local GC content (20 bp, x-axis). SNPs were divided into 5 groups according to their regional 200 bp GC content (columns) and by the mutation type (row). Gray dashed curves represent the 95% confidence intervals.
(TIF)

Figure S4 Divergence rates around microsatellites. Observed divergence rates (solid curves) and expected divergence rates (dashed curves, based on global genomic rates) versus the distance (x-axis) to the nearest A) (A)_6_ B) (AT)_3_ or (TA)_3_ C) (CA)_3_ or (AC)_3_ D) (GA_)3_ or (AG)_3_. Due to their strong enrichment around CEs, the sequence flanking e.g., CA repeats are highly implicated in conservation. As shown in Figure 4, proper normalization suggests that despite this, CA repeats are not more strongly enriched around CEs than other classes of tandem repeats.
(TIF)

Figure S5 A–B) Polymorphism patterns in human (A) and mouse (B) support non neutral evolution of GC content in mammals. Shown is the frequency of rare alleles p(minor allele<0.082) of different mutation types (rows) versus local (20 bp) GC content (X-axis). Analysis is stratified over regional (1 kb) GC content (columns of panels). Gray curves represent 95% binomial confidence intervals. While biased gene conversion or recombination intensities are known to be correlated with GC content and can thereby indirectly contribute to the association between GC content and the frequency of rare alleles, the data here suggest that small scale GC content is significantly linked with an increase in rare allele frequency independently of these effects. C) Large fraction of the genome is affected by conserved elements and associated structural sequences. Percentages of Exonic DNA (29.4 Mb, 24.4%), CEs (3.4 Mb, 2.8%) CE surrounding (26.7 Mb, 22.1%, distance <300 bp) and remaining DNA (60.9 Mb 50.5%). More than 33% of the non-exonic sequences are CE associated sequences.
(TIF)

Table S1 Enrichment of different epigenetic marks over CEs. Log2 ratios of averages of 315 analyzed epigenomic marks (column A) between CEs and randomized intervals of the same lengths (column B) and comparing to the regional levels (1 kb shift of the intervals) of the same marks (column C).
(XLS)

Table S2 Epigenomic CEs cluster xii lacks enrichment of epigenetic marks over. Similar to S1, but here enrichment of epigenomic marks are only for CE's within epigenomic cluster xii. The table shows that enrichments are significantly low comparing to overall enrichments.
(XLS)

Table S3 Exhaustive 6-mer screen around conserved elements. Frequency of each 6-mer within CEs and at 50 bp distance bins around conserved elements.
(XLS)

## References

[1] Mcvicker G, Gordon D, Davis C, Green P (2009) Widespread Genomic Signatures of Natural Selection in Hominid Evolution. PLoS Genet 5: e1000471 doi:10.1371/journal.pgen.1000471. 19424416 10.1371/journal.pgen.1000471 PMC2669884

[2] Raveh-Sadka T, Levo M, Shabi U, Shany B, Keren L, et al (2012) Manipulating nucleosome disfavoring sequences allows fine-tune regulation of gene expression in yeast. Nature genetics 44: 743–750 doi:10.1038/ng.2305. 22634752 10.1038/ng.2305

[3] Liu X, Lee C-K, Granek JA, Clarke ND, Lieb JD (2006) Whole-genome comparison of Leu3 binding in vitro and in vivo reveals the importance of nucleosome occupancy in target site selection. Genome research 16: 1517–28 doi:10.1101/gr.5655606. 17053089 10.1101/gr.5655606 PMC1665635

[4] Kaplan N, Moore IK, Fondufe-Mittendorf Y, Gossett AJ, Tillo D, et al (2008) The DNA-encoded nucleosome organization of a eukaryotic genome. Nature 458: 362–366 doi:10.1038/nature07667. 19092803 10.1038/nature07667 PMC2658732

[5] Tillo D, Hughes T (2009) G+C content dominates intrinsic nucleosome occupancy. BMC bioinformatics 10: 442 doi:10.1186/1471-2105-10-442. 20028554 10.1186/1471-2105-10-442 PMC2808325

[6] Valouev A, Johnson SM, Boyd SD, Smith CL, Fire AZ, et al (2011) Determinants of nucleosome organization in primary human cells. Nature 474: 516–20 doi:10.1038/nature10002. 21602827 10.1038/nature10002 PMC3212987

[7] Gaffney DJ, McVicker G, Pai AA, Fondufe-Mittendorf YN, Lewellen N, et al (2012) Controls of Nucleosome Positioning in the Human Genome. PLoS Genet 8: e1003036 doi:10.1371/journal.pgen.1003036. 23166509 10.1371/journal.pgen.1003036 PMC3499251

[8] Schuster-Böckler B, Lehner B (2012) Chromatin organization is a major influence on regional mutation rates in human cancer cells. Nature 488: 504–7 doi:10.1038/nature11273. 22820252 10.1038/nature11273

[9] Kundaje A, Kyriazopoulou-Panagiotopoulou S, Libbrecht M, Smith CL, Raha D, et al (2012) Ubiquitous heterogeneity and asymmetry of the chromatin environment at regulatory elements. Genome Research 22: 1735–1747 doi:10.1101/gr.136366.111. 22955985 10.1101/gr.136366.111 PMC3431490

[10] Warnecke T, Batada NN, Hurst LD (2008) The impact of the nucleosome code on protein-coding sequence evolution in yeast. PLoS Genet 4: e1000250 doi:10.1371/journal.pgen.1000250. 18989456 10.1371/journal.pgen.1000250 PMC2570795

[11] Washietl S, Machné R, Goldman N (2008) Evolutionary footprints of nucleosome positions in yeast. Trends in genetics: TIG 24: 583–7 doi:10.1016/j.tig.2008.09.003. 18951646 10.1016/j.tig.2008.09.003

[12] Kenigsberg E, Bar A, Segal E, Tanay A (2010) Widespread Compensatory Evolution Conserves DNA-Encoded Nucleosome Organization in Yeast. PLoS Comput Biol 6: e1001039 doi:10.1371/journal.pcbi.1001039. 21203484 10.1371/journal.pcbi.1001039 PMC3009600

[13] Adams MD (2000) The Genome Sequence of Drosophila melanogaster. Science 287: 2185–2195 doi:10.1126/science.287.5461.2185. 10731132 10.1126/science.287.5461.2185

[14] Roy S, Ernst J, Kharchenko PV, Kheradpour P, Negre N, et al (2010) Identification of functional elements and regulatory circuits by Drosophila modENCODE. Science (New York, N.Y.) 330: 1787–97 doi:10.1126/science.1198374. 10.1126/science.1198374 PMC3192495 21177974

[15] Nègre N, Brown CD, Ma L, Bristow CA, Miller SW, et al (2011) A cis-regulatory map of the Drosophila genome. Nature 471: 527–31 doi:10.1038/nature09990. 21430782 10.1038/nature09990 PMC3179250

[16] Sexton T, Yaffe E, Kenigsberg E, Bantignies F, Leblanc B, et al (2012) Three-Dimensional Folding and Functional Organization Principles of the Drosophila Genome. Cell 148: 458–72 doi:10.1016/j.cell.2012.01.010. 22265598 10.1016/j.cell.2012.01.010

[17] Andolfatto P (2005) Adaptive evolution of non-coding DNA in Drosophila. Nature 437: 1149–52 doi:10.1038/nature04107. 16237443 10.1038/nature04107

[18] Halligan DL, Keightley PD (2006) Ubiquitous selective constraints in the Drosophila genome revealed by a genome-wide interspecies comparison. Genome research 16: 875–84 doi:10.1101/gr.5022906. 16751341 10.1101/gr.5022906 PMC1484454

[19] Haddrill PR, Bachtrog D, Andolfatto P (2008) Positive and negative selection on noncoding DNA in Drosophila simulans. Molecular biology and evolution 25: 1825–34 doi:10.1093/molbev/msn125. 18515263 10.1093/molbev/msn125 PMC2734132

[20] Mackay TFC, Richards S, Stone EA, Barbadilla A, Ayroles JF, et al (2012) The Drosophila melanogaster Genetic Reference Panel. Nature 482: 173–8 doi:10.1038/nature10811. 22318601 10.1038/nature10811 PMC3683990

[21] Moshkin YM, Chalkley GE, Kan TW, Reddy BA, Ozgur Z, et al (2012) Remodelers organize cellular chromatin by counteracting intrinsic histone-DNA sequence preferences in a class-specific manner. Molecular and cellular biology 32: 675–88 doi:10.1128/MCB.06365-11. 22124157 10.1128/MCB.06365-11 PMC3266603

[22] Mavrich TN, Jiang C, Ioshikhes IP, Li X, Venters BJ, et al (2008) Nucleosome organization in the Drosophila genome. Nature 453: 358–62 doi:10.1038/nature06929. 18408708 10.1038/nature06929 PMC2735122

[23] Chachick R, Tanay A (2012) Inferring Divergence of Context-Dependent Substitution Rates in Drosophila Genomes with Applications to Comparative Genomics. Mol Biol Evol 29: 1769–80 doi: 10.1093/molbev/mss056. 22319143 10.1093/molbev/mss056

[24] Mewes HW, Albermann K, Bähr M, Frishman D, Gleissner A, et al (1997) Overview of the yeast genome. Nature 387: 7–65 doi:10.1038/42755. 10.1038/42755 9169865

[25] Aerts S, Thijs G, Dabrowski M, Moreau Y, Moor B De (2004) Comprehensive analysis of the base composition around the transcription start site in Metazoa. BMC genomics 5: 34 doi:10.1186/1471-2164-5-34. 15171795 10.1186/1471-2164-5-34 PMC436054

[26] Li L, Zhu Q, He X, Sinha S, Halfon MS (2007) Large-scale analysis of transcriptional cis-regulatory modules reveals both common features and distinct subclasses. Genome biology 8: R101 doi:10.1186/gb-2007-8-6-r101. 17550599 10.1186/gb-2007-8-6-r101 PMC2394749

[27] Thomas S, Li X-Y, Sabo PJ, Sandstrom R, Thurman RE, et al (2011) Dynamic reprogramming of chromatin accessibility during Drosophila embryo development. Genome biology 12: R43 doi:10.1186/gb-2011-12-5-r43. 21569360 10.1186/gb-2011-12-5-r43 PMC3219966

[28] Sabo PJ, Hawrylycz M, Wallace JC, Humbert R, Yu M, et al (2004) Discovery of functional noncoding elements by digital analysis of chromatin structure. Proceedings of the National Academy of Sciences of the United States of America 101: 16837–42 doi:10.1073/pnas.0407387101. 15550541 10.1073/pnas.0407387101 PMC534745

[29] Neph S, Vierstra J, Stergachis AB, Reynolds AP, Haugen E, et al (2012) An expansive human regulatory lexicon encoded in transcription factor footprints. Nature 489: 83–90 doi:10.1038/nature11212. 22955618 10.1038/nature11212 PMC3736582

[30] Thurman RE, Rynes E, Humbert R, Vierstra J, Maurano MT, et al (2012) The accessible chromatin landscape of the human genome. Nature 489: 75–82 doi:10.1038/nature11232. 22955617 10.1038/nature11232 PMC3721348

[31] Sabo PJ, Humbert R, Hawrylycz M, Wallace JC, Dorschner MO, et al (2004) Genome-wide identification of DNaseI hypersensitive sites using active chromatin sequence libraries. Proceedings of the National Academy of Sciences of the United States of America 101: 4537–42 doi:10.1073/pnas.0400678101. 15070753 10.1073/pnas.0400678101 PMC384782

[32] Tillo D, Kaplan N, Moore IK, Fondufe-Mittendorf Y, Gossett AJ, et al (2010) High nucleosome occupancy is encoded at human regulatory sequences. PLoS ONE 5: e9129 doi:10.1371/journal.pone.0009129. 20161746 10.1371/journal.pone.0009129 PMC2817738

[33] Asthana S, Roytberg M, Stamatoyannopoulos J, Sunyaev S (2007) Analysis of sequence conservation at nucleotide resolution. PLoS Comput Biol 3: e254 doi:10.1371/journal.pcbi.0030254. 18166073 10.1371/journal.pcbi.0030254 PMC2230682

[34] Walter K, Abnizova I, Elgar G, Gilks WR (2005) Striking nucleotide frequency pattern at the borders of highly conserved vertebrate non-coding sequences. Trends in genetics: TIG 21: 436–40 doi:10.1016/j.tig.2005.06.003. 15979195 10.1016/j.tig.2005.06.003

[35] Vavouri T, Walter K, Gilks WR, Lehner B, Elgar G (2007) Parallel evolution of conserved non-coding elements that target a common set of developmental regulatory genes from worms to humans. Genome biology 8: R15 doi:10.1186/gb-2007-8-2-r15. 17274809 10.1186/gb-2007-8-2-r15 PMC1852409

[36] Macdonald SJ, Long AD (2006) Fine scale structural variants distinguish the genomes of Drosophila melanogaster and D. pseudoobscura. Genome biology 7: R67 doi:10.1186/gb-2006-7-7-R67. 16872532 10.1186/gb-2006-7-7-r67 PMC1779558

[37] Schuettengruber B, Ganapathi M, Leblanc B, Portoso M, Jaschek R, et al (2009) Functional anatomy of polycomb and trithorax chromatin landscapes in Drosophila embryos. PLoS Biol 7: e13 doi:10.1371/journal.pbio.1000013. 19143474 10.1371/journal.pbio.1000013 PMC2621266

[38] Zeitlinger J, Zinzen RP, Stark A, Kellis M, Zhang H, et al (2007) Whole-genome ChIP-chip analysis of Dorsal, Twist, and Snail suggests integration of diverse patterning processes in the Drosophila embryo. Genes & development 21: 385–90 doi:10.1101/gad.1509607. 17322397 10.1101/gad.1509607 PMC1804326

[39] Haddrill PR, Charlesworth B, Halligan DL, Andolfatto P (2005) Patterns of intron sequence evolution in Drosophila are dependent upon length and GC content. Genome biology 6: R67 doi:10.1186/gb-2005-6-8-r67. 16086849 10.1186/gb-2005-6-8-r67 PMC1273634

[40] Galtier N, Bazin E, Bierne N (2006) GC-biased segregation of noncoding polymorphisms in Drosophila. Genetics 172: 221–8 doi:10.1534/genetics.105.046524. 16157668 10.1534/genetics.105.046524 PMC1456149

[41] Duret L, Arndt PF (2008) The impact of recombination on nucleotide substitutions in the human genome. PLoS Genet 4: e1000071 doi:10.1371/journal.pgen.1000071. 18464896 10.1371/journal.pgen.1000071 PMC2346554

[42] Berglund J, Pollard KS, Webster MT (2009) Hotspots of biased nucleotide substitutions in human genes. PLoS Biol 7: e26 doi:10.1371/journal.pbio.1000026. 19175294 10.1371/journal.pbio.1000026 PMC2631073

[43] Kimura M (1985) The role of compensatory neutral mutations in molecular evolution. Journal of Genetics 64: 7–19 doi:10.1007/BF02923549.

[44] Doniger SW, Fay JC (2007) Frequent gain and loss of functional transcription factor binding sites. PLoS Comput Biol 3: e99 doi:10.1371/journal.pcbi.0030099. 17530920 10.1371/journal.pcbi.0030099 PMC1876492

[45] Li Y, Vinckenbosch N, Tian G, Huerta-Sanchez E, Jiang T, et al (2010) Resequencing of 200 human exomes identifies an excess of low-frequency non-synonymous coding variants. Nature genetics 42: 969–72 doi:10.1038/ng.680. 20890277 10.1038/ng.680

[46] Stone EA (2012) Joint genotyping on the fly: Identifying variation among a sequenced panel of inbred lines. Genome Res 22: 966–74 doi:10.1101/gr.129122.111. 22367192 10.1101/gr.129122.111 PMC3337441

[47] Haddrill PR, Charlesworth B (2008) Non-neutral processes drive the nucleotide composition of non-coding sequences in Drosophila. Biology letters 4: 438–41 doi:10.1098/rsbl.2008.0174. 18505714 10.1098/rsbl.2008.0174 PMC2515589

[48] Ozdemir A, Fisher-Aylor KI, Pepke S, Samanta M, Dunipace L, et al (2011) High resolution mapping of Twist to DNA in Drosophila embryos: Efficient functional analysis and evolutionary conservation. Genome research 21: 566–577 doi:10.1101/gr.104018.109. 21383317 10.1101/gr.104018.109 PMC3065704

[49] Nussinov R (1986) Some guidelines for identification of recognition sequences: regulatory sequences frequently contain (T)GTG/CAC(A), TGA/TCA and (T)CTC/GAG(A). Biochimica et biophysica acta 866: 93–108. 3513842 10.1016/0167-4781(86)90106-5

[50] Eden E, Lipson D, Yogev S, Yakhini Z (2007) Discovering motifs in ranked lists of DNA sequences. PLoS Comput Biol 3: e39 doi:10.1371/journal.pcbi.0030039. 17381235 10.1371/journal.pcbi.0030039 PMC1829477

[51] Crocker J, Potter N, Erives A (2010) Dynamic evolution of precise regulatory encodings creates the clustered site signature of enhancers. Nature communications 1: 99 doi:10.1038/ncomms1102. 10.1038/ncomms1102 PMC2963808 20981027

[52] Iyer V, Struhl K (1995) Poly(dA:dT), a ubiquitous promoter element that stimulates transcription via its intrinsic DNA structure. The EMBO journal 14: 2570–9. 7781610 10.1002/j.1460-2075.1995.tb07255.x PMC398371

[53] Lusk RW, Eisen MB (2010) Evolutionary mirages: selection on binding site composition creates the illusion of conserved grammars in Drosophila enhancers. PLoS Genet 6: e1000829 doi:10.1371/journal.pgen.1000829. 20107516 10.1371/journal.pgen.1000829 PMC2809757

[54] Cai JJ, Macpherson JM, Sella G, Petrov DA (2009) Pervasive hitchhiking at coding and regulatory sites in humans. PLoS Genet 5: e1000336. 19148272 10.1371/journal.pgen.1000336 PMC2613029

[55] Birney E, Stamatoyannopoulos JA, Dutta A, Guigó R, Gingeras TR, et al (2007) Identification and analysis of functional elements in 1% of the human genome by the ENCODE pilot project. Nature 447: 799–816 doi:10.1038/nature05874. 17571346 10.1038/nature05874 PMC2212820

[56] Prendergast JGD, Semple CAM (2011) Widespread signatures of recent selection linked to nucleosome positioning in the human lineage. Genome research 21: 1777–87 doi:10.1101/gr.122275.111. 21903742 10.1101/gr.122275.111 PMC3205563

[57] Fullerton SM, Bernardo Carvalho A, Clark AG (2001) Local Rates of Recombination Are Positively Correlated with GC Content in the Human Genome. Molecular Biology and Evolution 18: 1139–1142. 11371603 10.1093/oxfordjournals.molbev.a003886

[58] White EJ, Emanuelsson O, Scalzo D, Royce T, Kosak S, et al (2004) DNA replication-timing analysis of human chromosome 22 at high resolution and different developmental states. Proceedings of the National Academy of Sciences of the United States of America 101: 17771–6. 15591350 10.1073/pnas.0408170101 PMC539744

[59] Crocker J, Tamori Y, Erives A (2008) Evolution acts on enhancer organization to fine-tune gradient threshold readouts. PLoS Biol 6: e263 doi:10.1371/journal.pbio.0060263. 18986212 10.1371/journal.pbio.0060263 PMC2577699

[60] Erives A, Levine M (2004) Coordinate enhancers share common organizational features in the Drosophila genome. Proceedings of the National Academy of Sciences of the United States of America 101: 3851–6 doi:10.1073/pnas.0400611101. 15026577 10.1073/pnas.0400611101 PMC374333

[61] Worth CL, Gong S, Blundell TL (2009) Structural and functional constraints in the evolution of protein families. Nature Reviews Molecular Cell Biology 10: 709–720. 19756040 10.1038/nrm2762

[62] Schones DE, Cui K, Cuddapah S, Roh T-Y, Barski A, et al (2008) Dynamic regulation of nucleosome positioning in the human genome. Cell 132: 887–98 doi:10.1016/j.cell.2008.02.022. 18329373 10.1016/j.cell.2008.02.022 PMC10894452

[63] Lieberman-Aiden E, Berkum NL van, Williams L, Imakaev M, Ragoczy T, et al (2009) Comprehensive mapping of long-range interactions reveals folding principles of the human genome. Science (New York, N.Y.) 326: 289–93 doi:10.1126/science.1181369. 10.1126/science.1181369 PMC2858594 19815776

[64] Tolhuis B, Palstra R-J, Splinter E, Grosveld F, Laat W de (2002) Looping and Interaction between Hypersensitive Sites in the Active β-globin Locus. Molecular Cell 10: 1453–1465. 12504019 10.1016/s1097-2765(02)00781-5

[65] Levine M (2010) Transcriptional enhancers in animal development and evolution. Current biology: CB 20: R754–63 doi:10.1016/j.cub.2010.06.070. 20833320 10.1016/j.cub.2010.06.070 PMC4280268

[66] Bantignies F, Roure V, Comet I, Leblanc B, Schuettengruber B, et al (2011) Polycomb-dependent regulatory contacts between distant Hox loci in Drosophila. Cell 144: 214–26 doi:10.1016/j.cell.2010.12.026. 21241892 10.1016/j.cell.2010.12.026

[67] Rhead B, Karolchik D, Kuhn RM, Hinrichs AS, Zweig AS, et al (2010) The UCSC Genome Browser database: update 2010. Nucleic acids research 38: D613–9 doi:10.1093/nar/gkp939. 19906737 10.1093/nar/gkp939 PMC2808870

[68] Langmead B, Salzberg SL (2012) Fast gapped-read alignment with Bowtie 2. Nature Methods 9: 357–359 doi:10.1038/nmeth.1923. 22388286 10.1038/nmeth.1923 PMC3322381

[69] Weber CM, Henikoff JG, Henikoff S (2010) H2A.Z nucleosomes enriched over active genes are homotypic. Nature structural & molecular biology 17: 1500–7 doi:10.1038/nsmb.1926. 10.1038/nsmb.1926 PMC3051840 21057526

[70] Gallo SM, Gerrard DT, Miner D, Simich M, Soye BDes, et al (2011) REDfly v3.0: toward a comprehensive database of transcriptional regulatory elements in Drosophila. Nucleic acids research 39: D118–23 doi:10.1093/nar/gkq999. 20965965 10.1093/nar/gkq999 PMC3013816

[71] Cohen NM, Kenigsberg E, Tanay A (2011) Primate CpG islands are maintained by heterogeneous evolutionary regimes involving minimal selection. Cell 145: 773–786. 21620139 10.1016/j.cell.2011.04.024

[72] The International HapMap Project (2003) Nature 426: 789–96. 14685227 10.1038/nature02168

[73] Keane TM, Goodstadt L, Danecek P, White MA, Wong K, et al (2011) Mouse genomic variation and its effect on phenotypes and gene regulation. Nature 477: 289–94 doi:10.1038/nature10413. 21921910 10.1038/nature10413 PMC3276836

