# Supplementary figures and images for: Drosophila Functional Elements Are Embedded in Structurally Constrained Sequences
Source: PLoS Genet. 2013 May 30;9(5):e1003512. doi: 10.1371/journal.pgen.1003512 (PMC3671938; doi:10.1371/journal.pgen.1003512)

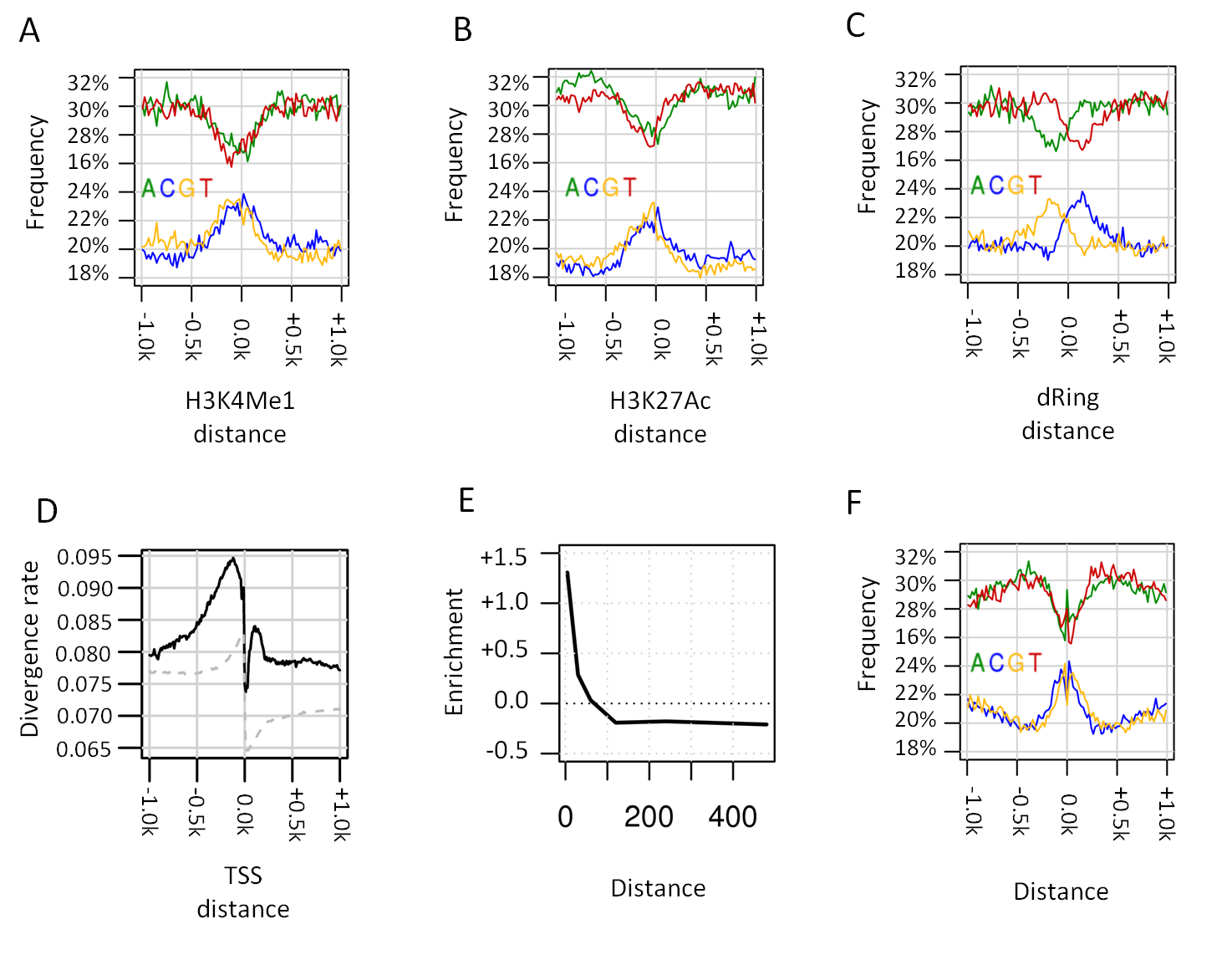

Supplement: Figure S1 — Fly regulatory elements are characterized by increased GC content and non-uniform evolutionary dynamics. A–C) Nucleotide composition around regulatory elements is shown versus the distance (x-axis) to the center of the nearest peak of A) H3K4Me1, B) H3K27Ac and C) dRing. The data showed that GC content is enriched over a region spanning ∼0.5 kb around regulatory elements of different classes. D) Increased substitution rates in fly promoters. Shown is substitution probability inferred from analysis of 12 drosophila species as a function of distance from the nearest TSS. While the divergence is lower near TSSs (more conserved), the 1 kb region upstream to TSS is highly diverged comparing to the background rate of evolution, reaching the maximum rate ∼150 bp upstream to TSS. E) Enrichment of transcription factor binding sites in CEs. Enrichment of REDfly experimentally validated transcription factor binding sites [70] is shown versus the distance from CE centers. Enrichment score is defined as the log2 ratio of the number of observed REDfly sites within a given distance range and the expected number of sites under uniform genomic distribution. F) Punctuated GC pattern in DHSs with weaker conservation. Shown are nucleotide frequencies versus the distance (x-axis) from the loci of minimum divergence within 1587/2652 DHSs that lack a CE given our current thresholds. To compute the pattern, we centered all elements on the positions with highest conservation score. Although these loci were not classified as CEs according to a stringent threshold, their punctuated GC pattern is significant. (TIF) [file pgen.1003512.s001.tif]

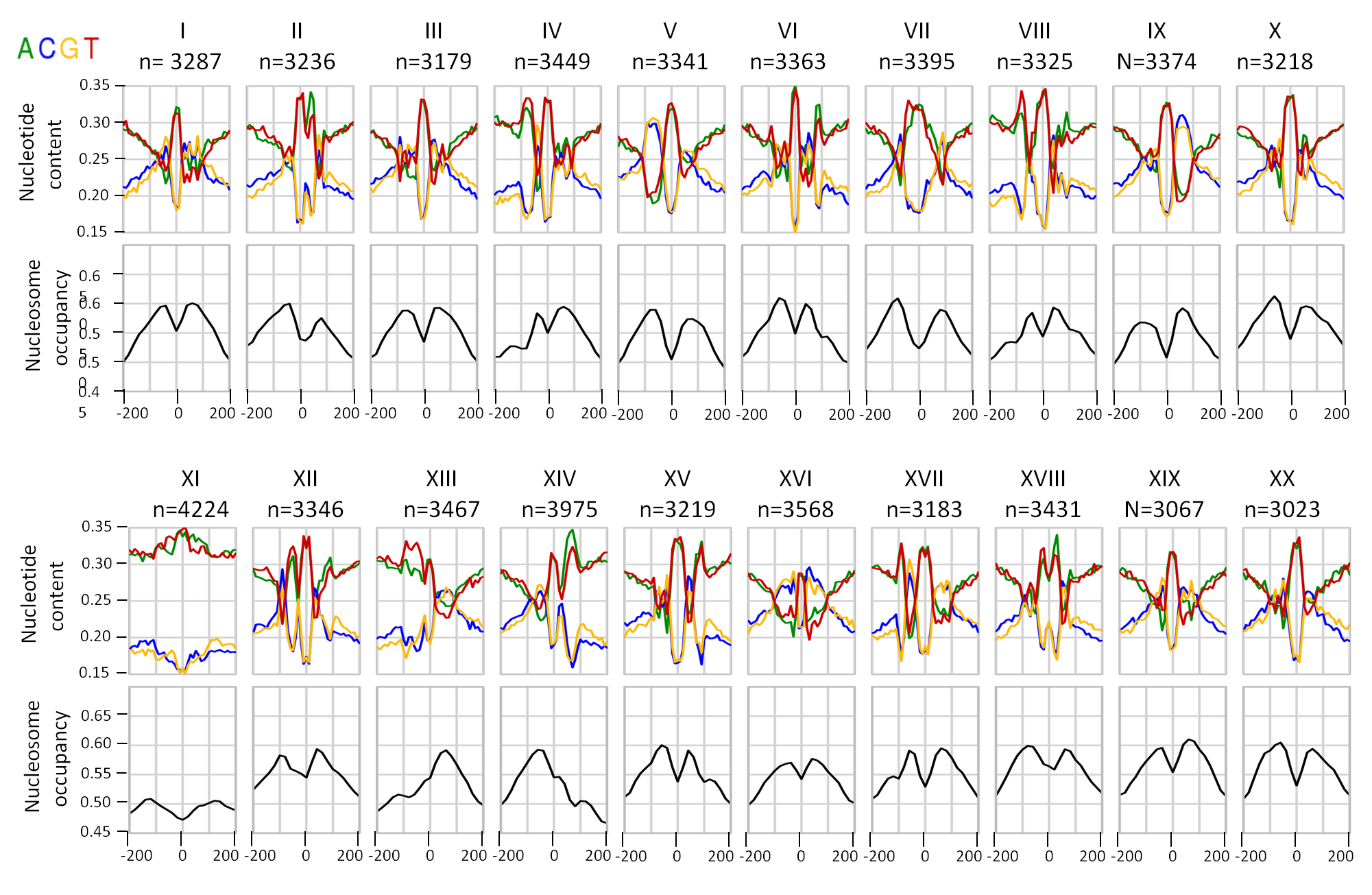

Supplement: Figure S2 — Classes of CE sequence compositions support the universality of the punctuated GC elevation pattern. CEs were clustered using k-means given their nucleotide composition patterns (Methods). Shown are nucleotide compositions and nucleosome occupancy profiles (y-axis) versus distance to the center of the nearest CE (x-axis) for 20 clusters inferred. We note that the clusters' nucleotide composition profile is varying in basal GC content and in symmetry, but that with the exception of one cluster (XI) all groups of CEs showed GC elevation in their proximity. (TIF) [file pgen.1003512.s002.tif]

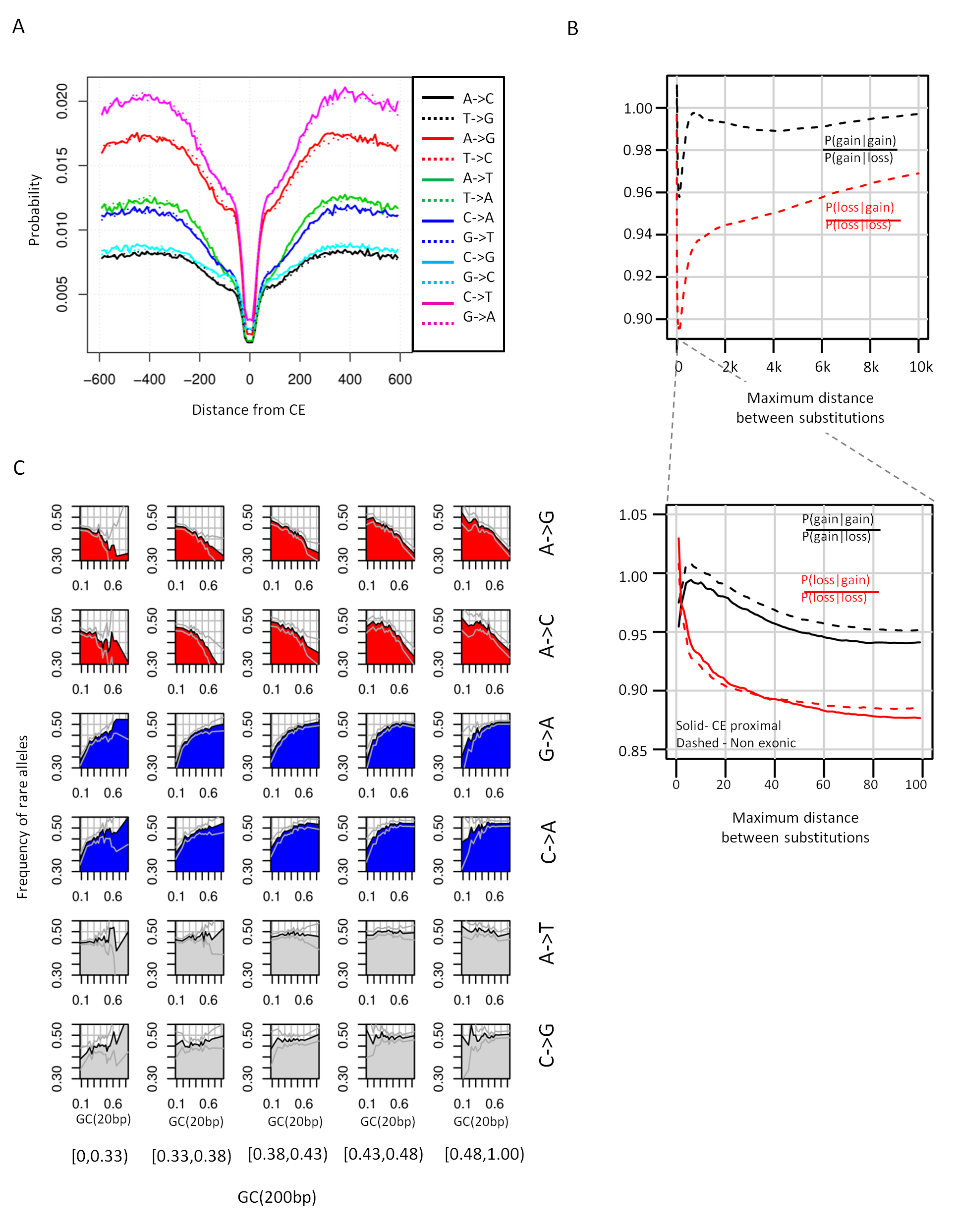

Supplement: Figure S3 — Evolutionary dynamics are associated with local GC content. A) Inferred substitution rates around CEs. Shown are substitution rates of all mutation types versus the distance from the nearest center of conserved element averaged over the melanogaster group lineages. Solid lines are color coded according to the substitution types A→C (black), A→G (red), A→T (green), C→A (blue), C→G (cyan), C→T (magenta). Dotted lines represent the complementing mutations. B) Association between GC gain and loss. We quantified the spatial coupling between GC gain and loss substitutions over the D. yakuba lineage, by estimating the rate of one type of substitution (GC gain in black, GC loss in red), conditioned on the existence of another type of substitution at a certain distance from it (X axis, Methods). The ratio between the two types of conditionings provides indication to the extent of coupling showing opposite trends for GC losing and gaining substitutions. Strong compensatory coupling was observed locally (at distances of less than 20 bp, see inset), while on the long range (e.g. >2 kb) we observed coupling of GC losing events (i.e. red curve is below 1). C) Stratification on regional GC content shows that fly allele frequency is strongly associated with local GC content. Shown is frequency of rare alleles (y-axis, see methods) against the local GC content (20 bp, x-axis). SNPs were divided into 5 groups according to their regional 200 bp GC content (columns) and by the mutation type (row). Gray dashed curves represent the 95% confidence intervals. (TIF) [file pgen.1003512.s003.tif]

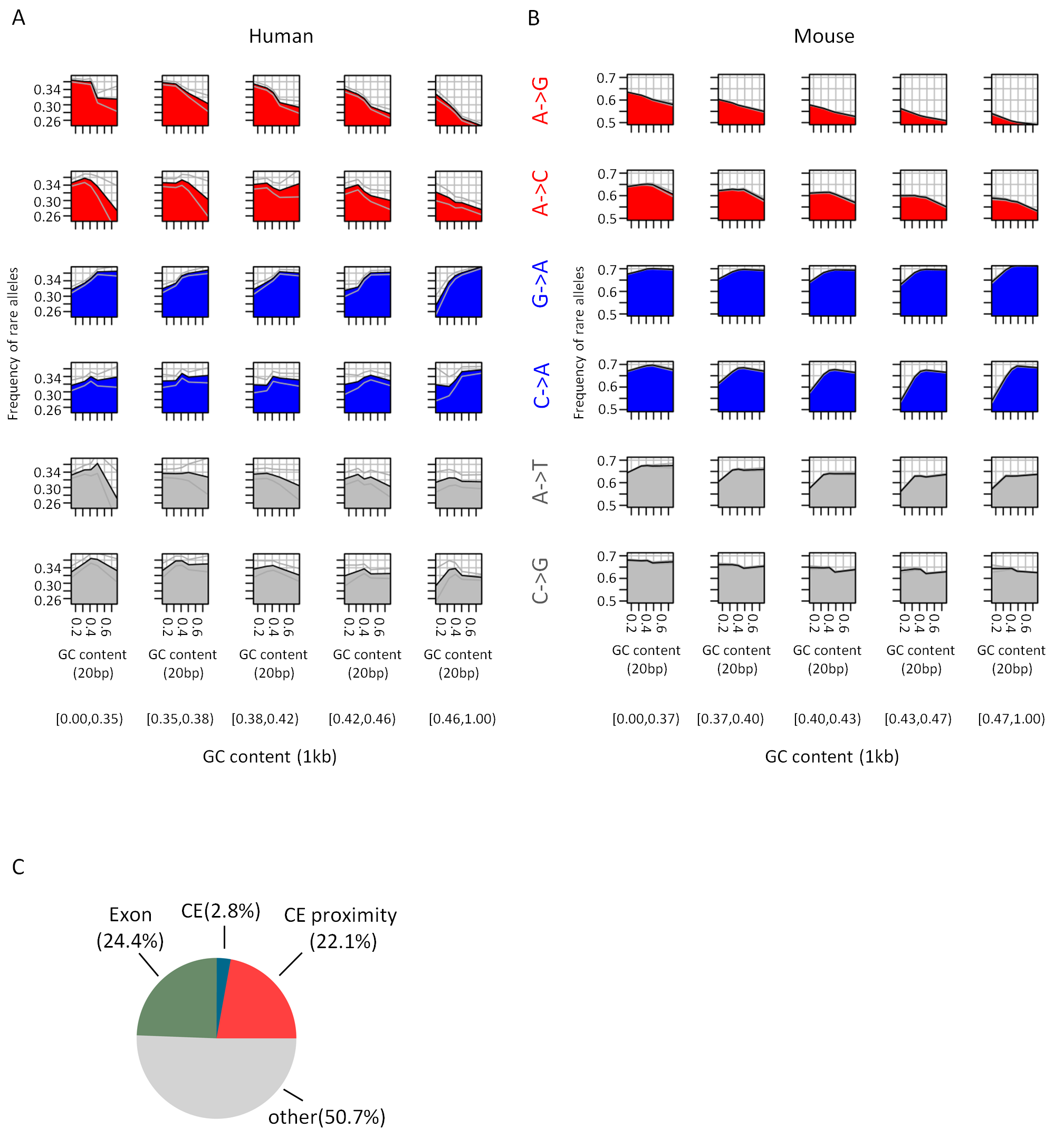

Supplement: Figure S5 — A–B) Polymorphism patterns in human (A) and mouse (B) support non neutral evolution of GC content in mammals. Shown is the frequency of rare alleles p(minor allele<0.082) of different mutation types (rows) versus local (20 bp) GC content (X-axis). Analysis is stratified over regional (1 kb) GC content (columns of panels). Gray curves represent 95% binomial confidence intervals. While biased gene conversion or recombination intensities are known to be correlated with GC content and can thereby indirectly contribute to the association between GC content and the frequency of rare alleles, the data here suggest that small scale GC content is significantly linked with an increase in rare allele frequency independently of these effects. C) Large fraction of the genome is affected by conserved elements and associated structural sequences. Percentages of Exonic DNA (29.4 Mb, 24.4%), CEs (3.4 Mb, 2.8%) CE surrounding (26.7 Mb, 22.1%, distance <300 bp) and remaining DNA (60.9 Mb 50.5%). More than 33% of the non-exonic sequences are CE associated sequences. (TIF) [file pgen.1003512.s005.tif]
